# Effect of Drawing Parameters on the Properties of Polypropylene/Inorganic Particles Composites by Solid-State Die Drawing

**DOI:** 10.3390/polym13223913

**Published:** 2021-11-12

**Authors:** Jianchen Cai, Jinyun Jiang, Qun Yang, Peng Cheng, Ke Chen, Suwei Wang

**Affiliations:** 1 College of Mechanical Engineering, Quzhou University, Quzhou 324000, China; cai198666@126.com (J.C.); jiangjinyun2@163.com (J.J.); 2 College of Mechanical and Electrical Engineering, Beijing University of Chemical Technology, Beijing 100029, China; honor0612@163.com (Q.Y.); cp1997888@163.com (P.C.); 3 National Special Superfine Powder Engineering Research Center of China, School of Chemistry and Chemical Engineering, Nanjing University of Science and Technology, Nanjing 210094, China; wangsw90@163.com

**Keywords:** solid-state die-drawing, PP, inorganic particles, mechanical properties

## Abstract

Die drawing is an effective method for improving the properties of polymer. In this work, polypropylene (PP)/inorganic particle composites were fabricated by a solid-state die drawing process to investigate the effects of drawing parameters, such as inorganic particles types, drawing temperature, and drawing speed, on the thermal properties, microstructure, and mechanical behavior of the drawn composites. The mechanical properties of the material were significantly improved through this processing method. For the drawn PP/inorganic particle composites with 45 wt% CaCO_3_, when the drawing speed was 2.0 m/min and the drawing temperature was 110 °C, the density of the drawn composites reached the lowest at 1.00 g/cm^3^. At this time, the tensile strength, flexural strength, and impact strength of the drawn composites were 128.32 MPa, 77.12 MPa, and 170.42 KJ/m^2^, respectively. This work provides a new strategy for the preparation of lightweight and high-strength PP-based composites, which have broad application prospects in the field of engineering and structural materials.

## 1. Introduction

PP is a semi-crystalline thermoplastic, which is one of the most widely used plastics in the world [1,2,3]. It has the characteristics of light weight, good heat and corrosion resistance, and easy processing, and it is widely used in packaging, electronic and electrical appliances, automobiles, textiles, and the food industry [4,5,6]. However, its low strength and modulus, poor impact resistance, and large molding shrinkage also make it difficult to be used as engineering and structural materials [7].

In order to achieve the high performance of PP, researchers have carried out a large number of works on the inorganic particles filling modification of PP [8,9,10]. By introducing rigid inorganic particles, it can not only improve the stiffness and heat resistance of polymer but also reduce the production cost. Talc powder (Talc) [11], calcium carbonate (CaCO_3_) [12,13], and glass beads [14] are generally used for filling modification, and some aspects of the performance of the PP composite modified by filling have been improved [15]. However, they are still insufficient as structural materials. The reason for this is that the flexible polymer chains of PP are entangled and not fully stretched, and the crystalline region forms a “spherulitic” structure [16,17,18]. The “spherulite” structure is surrounded by the amorphous phase and connected by the intergranular connecting tie molecules (TMs) [19,20]. The strength and modulus of PP are determined by the structure of TMs and the strength and modulus of the amorphous phase [21]. This leads to the fact that the potential mechanical strength of the material itself cannot be fully displayed.

The concept of consciously controlling the orientation and crystallization of polymer molecular chains in the molding process to improve the mechanical properties of polymers has long been put forward as so-called “self-reinforced composites” [22,23]. However, it is difficult for the molecular chains of polymers to form a highly oriented structure during traditional extrusion or the injection melting processing [24,25]. The molecular chains’ orientation of the polymer can be performed by introducing external stress during processing [26].

Solid-state die drawing of polymer was an effective way to achieve molecular chain disentanglement and orientation, which was first proposed by Coates and Ward [27,28]. During the die drawing, the polymer billet was heated to below the melting temperature (T_m_) and passed through die under the drawing load, where the flow channels converge. The molecular chains can be oriented under the combined action of axial load and the inner wall of the flow channel. A spherulite structure would deform and reorient along the drawing direction to form “string crystal” or “micro fiber crystal” structure [2].

The results of many studies have shown that composite material after inorganic filling had obvious mechanical performance improvement under the combined action of the drawing load at the free end [29,30]. Wu et al. [31] studied the properties of PP/multi-wall carbon nanotube composites under the die drawing method and showed that the tensile strength increased by 550% after drawing. Lin et al. [32] investigated the performance of porous tape prepared by solid-state drawn PP/Talc composites, and the results showed that the porosity increased with the increase in the drawing ratio. Rane et al. [30] studied the crystal orientation and microstructure development of PP/Talc composites under different tensile ratios. As far as the current research is concerned, there are few studies on the effect of drawing process parameters on the properties of PP/inorganic particle composites.

In this work, we used our self-designed die to carry out solid-state die drawing on composite materials to prepare lightweight and high-strength PP-based thermoplastic composites. CaCO_3_ and Talc of different contents were used to fill PP to study the influence of different types and contents of inorganic particle filler on the mechanical properties of composites, and a suitable material system was selected for the solid-state die-drawing research. In the process of drawing, the effects of different drawing temperatures and speeds on the properties of the final product were also included to study their influence on the mechanical behavior and micromorphology of materials. The research content of this work can guide the optimal material system and drawing process conditions for the preparation of inorganic particle-filled PP matrix composites by die drawing.

## 2. Materials and Methods

### 2.1. Materials

The polymer material used in this work was random copolymer polypropylene (6D83K, PP) that was purchased from Braskem Petrochemical Company, Sao Paulo, Brazil. The PP had a melt flow index (MFI) of 1.9 g/10 min at 230 °C/2.16 kg and a density of 0.90 g/cm^3^. The Talc with 1250 mesh was supplied by Shandong Xinruli Chemical Technology Co., Ltd., Jinan, China. The CaCO_3_, which was also 1250 mesh, was purchased from Zhongshan Laipeng New Material Co., Ltd., Zhongshan, China. The polymer and inorganic filler were dried at 100 °C for 4 h in blast drying oven before use. The polyethylene (PE) wax with a molecular weight of 2000–2500 was kindly provided by Qingdao Haihao Chemical Co., Ltd., Qingdao, China. The calcium stearate (CaSt) was purchased from Hebei Langfang Pengcai Fine Chemical Co., Ltd., Langfang, China. Particularly, PE wax and CaSt played the lubricating role in the PP/inorganic particle composite in order to promote extrusion and obtain a smooth surface. Table 1 presents the proportion of raw materials for solid-state die drawing in this work.

### 2.2. Preparation Procedure

**Figure 1:**
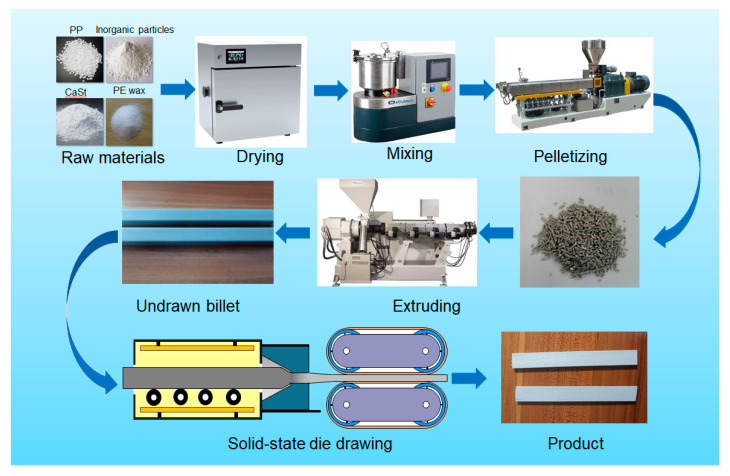
Diagram of the preparation of inorganic particle-filled PP composites by solid-state die drawing.

The PP, inorganic filler, and corresponding additives were weighed according to the proportion. The raw materials were dried and mixed evenly in a high-speed mixer. After mixing, the above raw materials were added to the twin-screw extruder for pelletizing and then were fed to the single-screw extruder for extruding. The inorganic particle-filled polypropylene composite billets, with a cross-section of 18 mm × 12 mm, were obtained by extrusion. Then, the billet was put into the preheating device and heated for 1 h before drawing. Under the action of the tractor, the drawn samples with a cross-section of 12.2 mm × 4 mm were obtained through the drawing die, and the drawing ratio was 4.4. The drawing ratio was the ratio of the billet size to the section at the die exit [32]. The preheating temperature was the same as the drawing temperature. The detailed preparation procedure is shown in Figure 1.

### 2.3. Mechanical Properties Testing

The mechanical properties of the samples were determined using a universal testing machine (Instron 5566, Instron Corporation, Boston, MA, USA). The samples were machined to testing size before measurement. The tensile strength of the samples was measured according to ISO 527-2012. The sample size was 150 mm × 12.2 mm × 4 mm, and the test speed was 10 mm/min. The flexural strength and modulus of the samples were tested based on ISO 178-2010. The sample size was 80 mm × 10 mm × 4 mm, and the span was 64 mm. The impact strength of the samples was tested by a pendulum impact testing machine (CEAST 9050, Instron Corporation, Boston, MA, USA), according to ISO 179-2010. The sample size was 80 mm × 10 mm × 4 mm, and the span was 70 mm. Before testing, a side-edge notch with a depth of 2 mm was machined on each sample. The pendulum speed was 3.5 m/s. Five specimens of each composite were tested, and the average value is reported.

### 2.4. Density Testing

The density of the billet and solid-state drawn samples were tested. The multifunctional solid density volume tester (AKD-310A) was used for density testing, which was purchased from Yangzhou Accurate Instrument Co., Ltd., Yangzhou, China. Five samples of each composites were measured to calculate the average as reliable results.

### 2.5. Thermal Properties Measurement

The Vicat softening temperature (VST) of the samples was measured by using a thermal deformation Vicat softening point temperature determination kit (ZB-909B, Jiangsu Zhengrui Taibang Electronic Technology Co., Ltd., Yangzhou, China) following ISO 306-2013. The size of the rectangular specimen was 80 mm × 10 mm × 4 mm, and the heating rate was 50 °C/h.

### 2.6. Scanning Electron Microscopy

Fractured surfaces of the prepared samples were observed after fracturing the samples along the drawing direction in liquid nitrogen. The cross-section of the sample was treated by spraying gold, then a scanning electron morphology (SEM) study was carried out using an electron microscope (S-4700, Hitachi, Tokyo, Japan).

## 3. Results and Discussion

### 3.1. Comparison of the Composites’ Properties before and after Drawing

**Figure 2:**
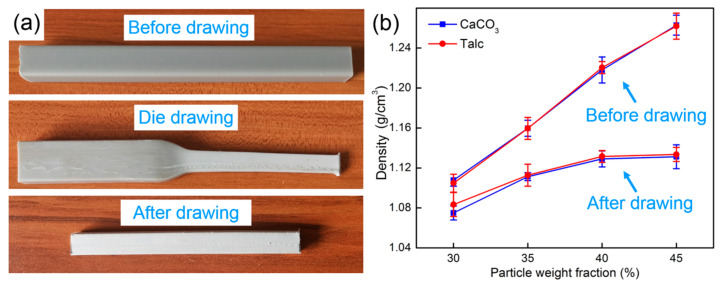
(**a**) PP/inorganic particle composites before, during, and after the drawing; (**b**) densities of the PP/inorganic particle composites before and after drawing.

Solid-state die drawing is an effective method for enhancing the mechanical properties of polymer materials. CaCO_3_ and Talc with different contents were used to investigate the effect of the content on the properties of the drawn composites. Figure 2a presents the PP/inorganic particle composite samples before, after drawing, and during die drawing. During the drawing process, the color of the sample gradually became lighter and finally changed from the original dark gray to light white. It seemed like the PP and the inorganic filler in the composites were pulled apart, which is illustrated by the following results.

The inorganic particle content was a significant factor affecting the properties of the composites. Under the conditions of a given drawing rate of 2 m/min and a 140 °C drawing temperature, the properties of PP/inorganic particle composites with different inorganic particle contents before and after drawing were studied. Figure 2b shows the densities of PP/inorganic particle composites before and after drawing. It can be seen from the figure that with the increase of inorganic particles content, the densities of PP/Talc and PP/CaCO_3_ composites before and after drawing were gradually increasing. After drawing, the density of the composites had decreased, and the more the content of inorganic particles, the greater the decrease in density. When the inorganic particles content reached 45 wt%, the density of PP/CaCO_3_ composites and PP/Talc composites decreased by 10.4% and 10.2%, respectively. The lowest density of the drawn composites was PP/CaCO_3_ composites and can reach 1.07 g/cm^3^.

### 3.2. Effect of Inorganic Particle Contents

**Figure 3:**
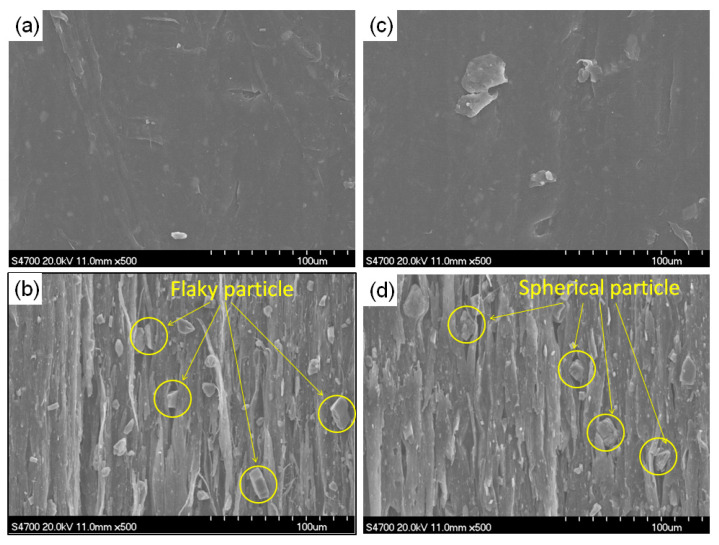
SEM micrograph of the composites: (**a**) PP/Talc composites before drawing; (**b**) PP/Talc composites after drawing; (**c**) PP/CaCO_3_ composites before drawing; (**d**) PP/CaCO_3_ composites after drawing.

Figure 3a–d present the SEM micrograph of PP/CaCO_3_ composites and PP/Talc composites before and after drawing, respectively. It can be seen that the orientation structure of PP was indeed formed after solid-state die drawing and micropores were also formed in the both PP/Talc and PP/CaCO_3_ composites. Inorganic particles played role in inducing micropore forming in the composites. The micropore size of PP/CaCO_3_ composites was relative larger than that of PP/Talc composites after drawing, owing to the spherical particle morphology of CaCO_3_ and flaky particle morphology of Talc. This also explained why the density of the PP/CaCO_3_ composites was lower than that of PP/Talc composites after drawing.

**Figure 4:**
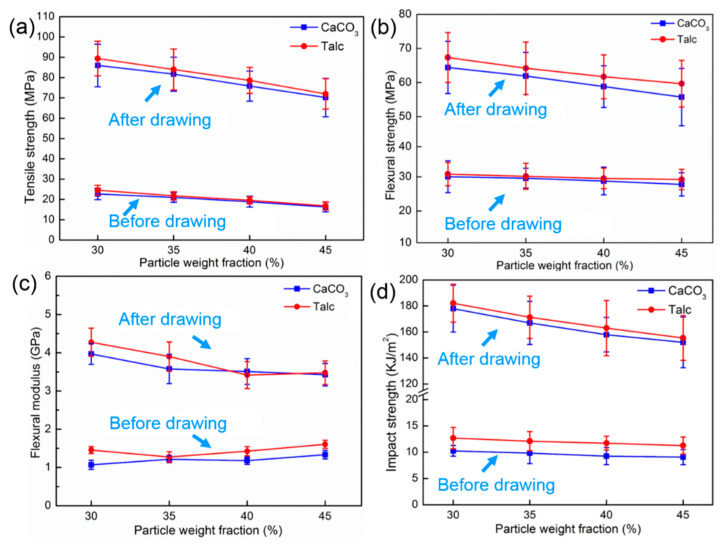
Mechanical properties of composites with different particle contents: (**a**) tensile strength; (**b**) flexural strength; (**c**) flexural modulus; (**d**) impact strength.

The mechanical behaviors of PP/inorganic particle composites with different inorganic particle contents before and after drawing are shown in Figure 4. It can be seen from Figure 4a that with the increase in inorganic particle content, the tensile strength of PP/inorganic particle composites before and after drawing showed the same downward trend. When the inorganic particle content was 30 wt%, the tensile strength of the undrawn PP/Talc and PP/CaCO_3_ composites were only 24.53 and 22.69 MPa, respectively. After drawing, the tensile strength of the PP/Talc and PP/CaCO_3_ composites reached 89.40 and 85.97 MPa, respectively, which were 264.5% and 219.6% higher than the tensile strengths of the undrawn samples, respectively. The flexural properties of the samples showed the same downward trend as the tensile strength, shown in Figure 4b,c. But the difference was that the flexural strength of the PP/Talc composites after drawing was slightly higher than that of the PP/CaCO_3_ composites. For a content of 45 wt% inorganic particles, the flexural strength of the PP/Talc composites was 59.65 MPa, which was 3.94 MPa higher than that of the PP/CaCO_3_ composites. The value of the flexural modulus was not much different; for 45 wt% inorganic particles, the PP/Talc and PP/CaCO_3_ composites after drawing reached 3.48 and 3.43 GPa, respectively. In addition, it was undeniable that the flexural properties of the samples were greatly improved. Compared with the samples before drawing, the flexural strength and the modulus of the drawn samples with 45 wt% content were nearly doubled and tripled. The comparison of the impact strength of the samples before and after drawing is also shown in Figure 4d. Before drawing, there were almost no differences in the impact strength under different particle contents; however, both the PP/Talc and PP/CaCO_3_ composites showed a downward trend after drawing. The impact strength of the drawn PP/Talc composites was slightly higher than that of the drawn PP/CaCO_3_ composites, which was due to the toughening effect of the Talc’s flake-shaped structure.

### 3.3. Effect of Drawing Temperature

The drawing temperature was also a key process parameter. PP/Talc and PP/CaCO_3_ composites with a 45 wt% inorganic particle content were chosen in order to study the properties of high filled composites under different drawn temperatures. The drawing rate was set as 2.0 m/min.

**Figure 5:**
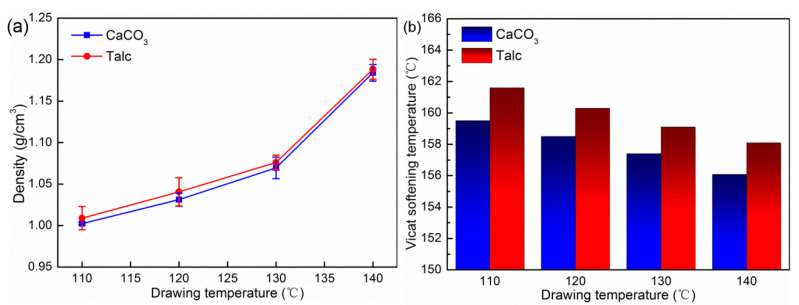
(**a**) Density of PP/inorganic particle composites with different drawing temperatures; (**b**) Vicat softening temperature of PP/inorganic particle composites with different drawing temperatures.

Figure 5 shows the density and Vicat softening temperature of the PP/Talc and PP/CaCO_3_ composites after drawing under different drawing temperatures. It can be seen that with the continuous increase in temperature, the density of the PP/Talc and PP/CaCO_3_ composites after drawing gradually increased. When the drawing temperature was 110 °C, the density of the PP/Talc and PP/CaCO_3_ composites after drawing reached the lowest, which were 1.01 g/cm^3^ and 1.00 g/cm^3^, respectively. The lower drawing temperature can promote a larger scale of debonding between PP and inorganic particles and reduce the density. Figure 5b shows the Vicat softening temperature of the composites with different drawing temperatures. The Vicat softening temperature of the composites decreased with the increase in the drawing temperature. In addition, the Vicat softening temperature of the PP/Talc composites was significantly higher than that of the PP/CaCO_3_ composites. Since high drawing temperatures significantly increased the activity of the PP molecular chain in the drawn composites and weakened the molecular chain orientation and the regular arrangement of resin molecular chains in the composites, it led to a decrease in the heat resistance of the drawn composites.

**Figure 6:**
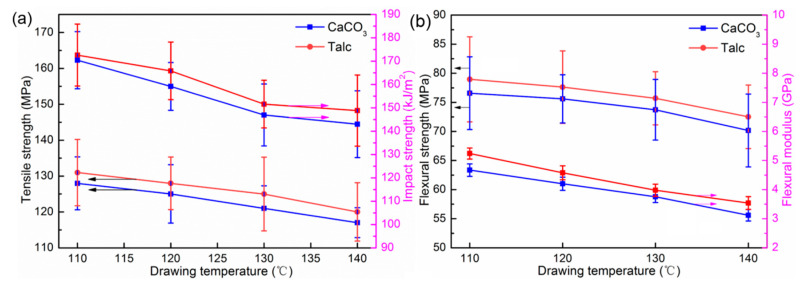
The mechanical properties of the drawn PP/Talc and PP/CaCO_3_ composites with differ-ent drawing temperatures: (**a**) tensile strength and impact strength; (**b**) flexural strength and flex-ural modulus.

The mechanical properties of drawn PP/Talc and PP/CaCO_3_ composites with different drawing temperatures are presented in Figure 6. As the drawing temperature increased, the tensile strength and impact strength of the drawn composites gradually decreased. When the drawing temperature was 110 °C, the tensile strength of the drawn PP/Talc and PP/CaCO_3_ composites were 131.12 and 128.32 MPa, which were approximately 7.71 times and 7.76 times higher than that of the undrawn composites, respectively. While the impact strength of the drawn PP/Talc and PP/CaCO_3_ composites showed the same downward trend, reaching the highest value at 110 °C, 172.61 kJ/m^2^, and 170.42 kJ/m^2^, respectively. The flexural properties of the drawn composites are shown in Figure 6b. From 110 to 140 °C, the flexural modulus of the drawn PP/Talc and PP/CaCO_3_ composites decreased by 29.5% and 30.4%, while the flexural strength decreased by 7.8% and 7.5%. Thus, with the increase in the drawing temperature, the effect on the flexural strength of the PP/Talc and PP/CaCO_3_ composites was not obvious compared with that on the flexural modulus.

The above results of the mechanical behavior indicate that as the drawing temperature increased, the tensile strength, flexural strength, and impact strength of the drawn composites showed a decreasing trend. Since there was a higher drawing temperature, there was stronger activity of the molecular chains inside the drawn composites; this did not promote the complete stretching of coiled molecular chains into linear molecular chains. The orientation degree of the molecular chain was reduced, resulting in the reduction of fiber structure in the composites along the drawing direction, which weakened the mechanical properties of the composites. Furthermore, the higher the temperature, the better the intermolecular compactness of the composites after converging and drawing through the die. Therefore, the stress easily concentrated in the dense molecular structure, causing stress cracking and developing a destructive cracking trend, resulting in the sample’s fracture and reduction in impact strength [26,33,34]. Thus, the impact strength of the composites after drawing gradually decreased.

**Figure 7:**
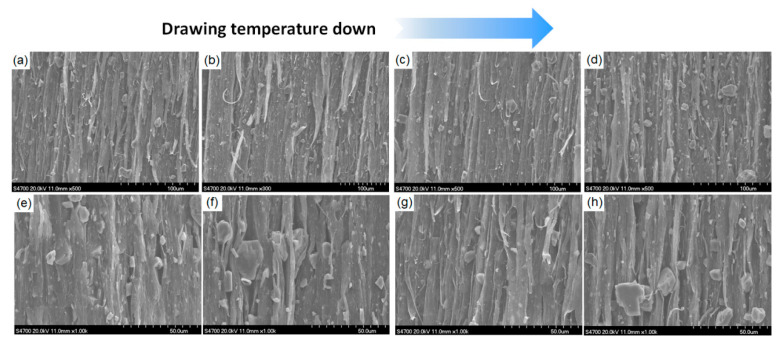
SEM micrographs of drawn composites: (**a**) PP/Talc composites at 110 °C; (**b**) PP/Talc composites at 130 °C; (**c**) PP/Talc composites at 120 °C; (**d**) PP/Talc composites at 110 °C; (**e**) PP/CaCO_3_ composites at 140 °C; (**f**) PP/CaCO_3_ composites at 130 °C; (**g**) PP/CaCO_3_ composites at 120 °C; (**h**) PP/CaCO_3_ composites at 110 °C.

The micromorphology of the PP/inorganic particle composites prepared under different drawing temperatures is shown in Figure 7. It can be observed that the lower the drawing temperature, the larger the micropore size under the micropore effect in the composites. This was because with the gradual decrease in the drawing temperature, the debonding effect between PP and inorganic particles was more obvious, resulting in an increase in the number of micropores in the drawn composites and in the length of the micropores along the drawing direction. This can also explain the trend seen in Figure 5a, that is, the density of the drawn composites decreased significantly with a decrease in the drawing temperature.

### 3.4. Effect of Drawing Speed

Drawing speed was another key process parameter in the solid-state die drawing. The undrawn composites with CaCO_3_ and Talc contents of 45 wt% were selected, and the drawing temperature was set to 140 °C. The changes in the density, mechanical behavior, and microstructure of the PP/inorganic particle composites at different drawing speeds were investigated.

**Figure 8:**
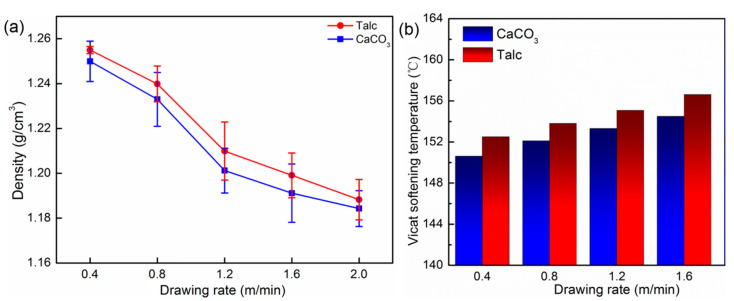
(**a**) Density of PP/inorganic particle composites with different drawing speeds; (**b**) the Vicat softening temperature of PP/inorganic particle composites with different drawing speeds.

The density and Vicat softening temperature of the PP/CaCO_3_ and PP/Talc composites under different drawing speeds are shown in Figure 8. It can be obtained from Figure 8a that as the drawing speed increased, the density of the drawn composites greatly decreased. When the drawing speed was 2 m/min, the density of the PP/CaCO_3_ and PP/Talc composites was at the minimum, which were 1.18 and 1.19 g/cm^3^, respectively. Since an increase in the drawing speed, which can promote the generation of voids in the composite material, and a higher speed can accelerate the debonding of inorganic particles from the PP matrix, it thereby reduces the density. The Vicat softening temperature of the composites rose with the increase in the drawing speed, and the Vicat softening temperature of the drawn PP/Talc composites was slightly higher than that of the PP/CaCO_3_ composites. This was because the molecular chains of the PP composite material were highly oriented with the increase in the drawing speed, and the fiber bundles generated during the orientation process could resist the pressure during the Vicat softening temperature test and then showed an increase in the Vicat softening temperature.

**Figure 9:**
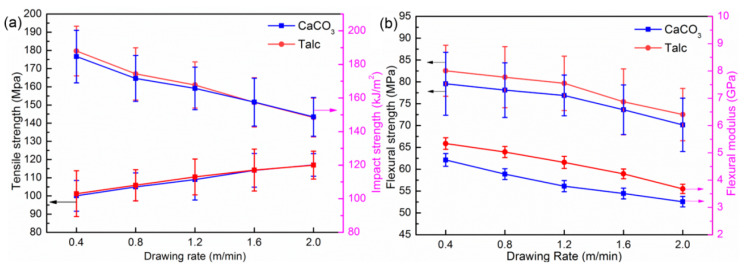
The mechanical properties of the drawn PP/Talc and PP/CaCO_3_ composites with differ-ent drawing rates: (**a**) tensile strength and impact strength; (**b**) flexural strength and flexural modulus.

The mechanical behaviors of drawn PP/CaCO_3_ and PP/Talc composites at different drawing speeds are shown in Figure 9. It can be noted from Figure 9a that as the drawing speed increased, the impact strength of the PP/inorganic particle composites decreased, while the tensile strength along the drawing direction showed the opposite trend. When the drawing speed was 0.4 m/min, the maximum impact strength of the PP/CaCO_3_ and PP/Talc composites were 180.1 and 177.51 KJ/m^2^, respectively. At this time, the tensile strength reached the minimum: 99.82 MPa and 101.64 MPa, respectively. The maximum tensile strength of the drawn PP/CaCO_3_ and PP/Talc composites appeared when the drawing speed was 2 m/min, which were 120.16 and 121.04 MPa, respectively. The impact strength was the minimum. The flexural properties of the drawn composites at different drawing speeds are shown in Figure 9b. The flexural strength and flexural modulus had the same change trend with the increase in drawing speed. When the drawing speed was 0.4 m/min, the flexural strength of the drawn PP/CaCO_3_ and PP/Talc composites reached the maximum, which were 86.46 MPa and 79.41 MPa, respectively. At this time, the flexural modulus was also at the maximum. Then, with the increase in drawing speed, the flexural strength and flexural modulus were weakened.

These changes in mechanical behavior can be attributed to the higher crystallinity and molecular chain orientation at higher drawing speeds. The flexural properties of the composites at different traction rates are shown in Figure 9b. The flexural strength and flexural modulus of the PP/CaCO_3_ and PP/Talc composites both decreased with the increase in the drawing speed. As the drawing speed increased, the density of the drawn composites decreased. The increase in the number of internal micropores led to the decrease in the flexural properties of the drawn composites.

**Figure 10:**
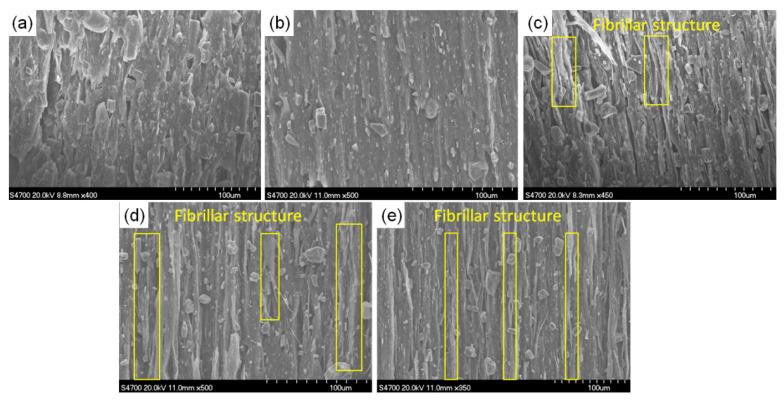
SEM micrographs of the PP/Talc composites under different drawing speeds: (**a**) PP/Talc composites at 0.4 m/min; (**b**) PP/Talc composites at 0.8 m/min; (**c**) PP/Talc composites at 1.2 m/min; (**d**) PP/Talc composites at 1.6 m/min; (**e**) PP/Talc composites at 2.0 m/min.

It can be observed that as the drawing speed increased, the fiber bundles inside the composites increased as illustrated in Figure 10 for the microstructure of the drawn PP/Talc composites. This indicated a boost in the orientation of the molecular chains within the material that was similar to the effect of the drawing temperature, where an increase in orientation could substantially improve the mechanical properties of the material after drawing. In addition, the debonding effect of the micropores from the PP matrix was obvious, and the number of micropores increased significantly, and the length along the fiber orientation direction increased with the rise of the drawing speed, thus reducing the density of composites.

**Figure 11:**
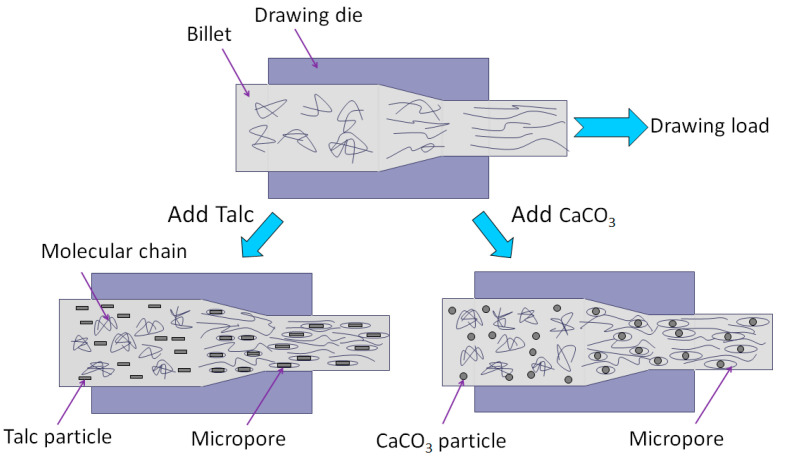
Schematic diagram of solid-state die drawing molding of CaCO_3_ or Talc filled PP composites.

The principle of processing CaCO_3_ or Talc filled PP composites by solid-state die drawing method is shown in Figure 11, which can well explain the process of micropore formation, molecular chain orientation, and fibrous structure formation during the drawing process. However, in this work, the die drawing behaviors of the PP/inorganic particle composites under different drawing ratios were not studied, only the die with a drawing ratio of 4.4 was used for investigation. The correlation mechanism between the drawing ratio and the mechanical properties of the PP/inorganic particle composites is our next focus of research.

## 4. Conclusions

In this work, the effects of different drawing temperatures, drawing speeds, inorganic particle contents and types on the mechanical behavior and micromorphology of drawn PP/inorganic particle composites were investigated with the solid-state die drawing method. The following conclusions were drawn:

(1) Solid-state drawing promoted density reduction, and the value of the density reduction was larger as the content of inorganic particles increased. The largest decrease was when the content of inorganic particles was 45%, and the density decreased by 10.4%. Compared with before drawing, the tensile strength, flexural strength, flexural modulus, and impact strength were all significantly enhanced, and the best was when the content of Talc was 30%, respectively, 89.97 MPa, 68.42 MPa, 4.26 GPa, and 182.62 KJ/m^2^;
(2) The increase in tensile temperature did not promote the formation of a fibrous structure of PP in the PP/inorganic particle composites, resulting in the deterioration of the properties of the composites. When the drawing temperature was 110 °C, the minimum density was 1.00 g/cm^3^, and the Vicat softening temperature was also the highest. The tensile strength, flexural strength, and impact strength were 131.12 MPa, 78.95 MPa, and 172.61 KJ/m^2^, respectively;
(3) The increase in the drawing speed promoted the generation of micropores in the composites and induced particle debonding. The Vicat softening temperature also increased with the increase in drawing speed. The tensile strength enhanced with the increase in drawing speed. When the drawing speed was 2 m/min, the tensile strength was 121.04 MPa, while the increase in the drawing speed weakened the flexural performance and impact strength.

## Figures and Tables

**Table 1:** Proportion of raw materials for solid-state die drawing.

Samples	PP (wt%)	CaCO_3_ (wt%)	Talc (wt%)	PE Wax (wt%)	CaSt (wt%)
Ca-30	68.5	30	-	1%	0.5%
Ca-35	63.5	35	-	1%	0.5%
Ca-40	58.5	40	-	1%	0.5%
Ca-45	53.5	45	-	1%	0.5%
Ta-30	68.5	-	30	1%	0.5%
Ta-35	63.5	-	35	1%	0.5%
Ta-40	58.5	-	40	1%	0.5%
Ta-45	53.5	-	45	1%	0.5%
